# Existing knowledge on Zn status biomarkers (1963–2021) with a particular focus on FADS1 and FADS2 diagnostic performance and recommendations for further research

**DOI:** 10.3389/fnut.2022.1057156

**Published:** 2023-01-12

**Authors:** Marija Knez, Erick Boy

**Affiliations:** ^1^Center of Research Excellence in Nutrition and Metabolism, Institute for Medical Research, National Institute of the Republic of Serbia, University of Belgrade, Belgrade, Serbia; ^2^HarvestPlus, International Food Policy Research Institute, Washington, DC, United States

**Keywords:** Zn biomarkers, plasma/serum Zn, LA/DGLA, desaturases, thymulin, dietary Zn intake

## Abstract

The role of Zn in human health was discovered 60 years ago, and despite remarkable research efforts, a sufficiently sensitive and specific biomarker of Zn status is still lacking. Plasma/serum Zn, currently the best available and most accepted population Zn status indicator, responds well to severe Zn deficiency, yet, mild to moderate Zn deficiency states usually remain unrecognized. Identifying early-stage Zn deficiency requires additional robust markers of Zn status. This paper discusses the sensitivity, specificity, and responsiveness of plasma Zn concentrations to Zn interventions. It describes the biochemical and dietary basis for the causal association between Zn and fatty acid desaturases activity, FADS1 and FADS2, based on data collected through studies performed in animals and/or humans. The influence of potential confounders and covariates on the observed relationships is considered. Additional potential Zn biomarkers are discussed and suggestions for further research in this area are provided.

## Plasma/serum Zn concentrations–the best available and accepted biomarker of Zn status in humans?

The story of Zn began 60 years ago when the role of Zn in human health was first recognized and since then, remarkable progress has been made in the understanding of Zn biochemistry, the biological role of Zn, clinical manifestations of Zn deficiency, and beneficial therapeutic impacts of Zn supplementation. Plasma Zn concentration (PZC) was measured for the first time in 1963 by the dithizone technique together with 24-h urine, hair, and red blood cell Zn concentrations (1).

A couple of years later, in 1965, the original technique for measuring Zn concentrations in plasma and blood cells, so-called atomic absorption spectrophotometry (AAS), was introduced (2). Ever since PZC measured by AAS has been widely employed as a biomarker of Zn deficiency, and it is still, despite its apparent disadvantages, commonly considered, as the best available biomarker for estimating Zn status in humans (3–5).

Both, PZC and serum Zn concentration (SZC) are regarded as valid estimates of Zn status.

The terms are used interchangeably, in this review they are both identified as “PZC”.

PZC cutoffs are established for various age and sex groups (6).

The efficacy of zinc interventions is frequently assessed using PZCs as a biomarker of Zn status in humans. PZC responds to severe Zn deficiency with clinical Zn manifestations, changes during Zn supplementation, and adapts to alterations in whole-body Zn balance (5). Clinical signs of Zn deficiency are developing progressively with reduced PZC, there is a clear direct association between the two parameters. Severe dietary Zn depletion, less than 1 mg Zn/day, produces a prompt and marked decline in PZC (7). While this is the case over a short period of time, homeostatic mechanisms maintain PZC within the physiological range, so the levels are not sustained over a prolonged period (4). PZC responds consistently to Zn supplementation, it reacts rapidly, within 5–10 days in all population groups, even to very low additional intakes of approximately 2 mg Zn/day (3, 4).

Zn supplementation increases PZC and after the withdrawal of Zn supplementation, the PZC returns to baseline levels within one to two weeks (5). PZC predicts a growth response to Zn supplementation only when the initial mean PZC is low enough indicating a moderate to severe Zn deficiency (5).

At the population level, PZC has responded to dietary manipulation in all population groups, and equally in depletion and supplementation studies in apparently healthy individuals (3, 5, 8). Every doubling of Zn intake contributes to a 6% difference in PZC (4). Yet, at the individual level, the association between PZC and Zn intakes is not that strong or consistent (5, 9).

For example, similar PZC was measured with intakes of 2.8 mg/kg as well as with 40 mg/kg, showing the inability of PZC to reliably represent dietary Zn intakes (9, 10). Complexities in evaluating an individual’s typical dietary Zn intake, Zn bioavailability, and Zn absorption, physiological states, presence and stage of inflammation, and differences in Zn absorption and Zn metabolism depending on the provision routes (i.e., Zn provided as supplement with or between meals vs. Zn given with various types of foods) make the process of assessing an individual’s Zn status by merely using PZC even more challenging (11). PZC remains normal even when disrupted Zn determined immunological processes are present (12). PZC changes less effectively in response to moderate modifications in dietary Zn intakes (3–5 mg Zn/day); 24 weeks of Zn restrictions are needed for the changes in PZC (13).

A meta-analysis of high-quality studies on the association between PZC and dietary Zn intake demonstrated a high degree of discrepancy in all population cohorts (5). No association of serum Zn with dietary or supplemental Zn intakes was seen in the NHANES study even though 8% of participants had serum Zn levels below the cut-off for Zn deficiency (14). No changes in PZC were seen after the consumption of Zn biofortified grains and foods either in children or adults (15–18). Additionally, PZC does not respond to short-term exposure to Zn fortified foods. However, certain functional changes may still occur without the changes in PZC, and this demands further investigation.

Furthermore, there is substantial interindividual variability in the way PZC respond to dietary Zn changes. Time of consumption of a meal, diurnal variation, sex, age, pregnancy, food intake, contraceptive use, hormones, and some drugs contribute to inconsistencies in PZC (5, 19). PZC varies by up to 20% during the day, predominantly due to meal consumption, and time of day (19–21). Generally, food intake produces a decline in PZC (20).

The highest PZC is measured early in the morning after an overnight fast, before breakfast, and then the levels gradually decrease for several hours after food intake and rise again prior to the next meal (21). PZC reacts to several physiological and pathological conditions, PZC is decreased during pregnancy and intense physical activity, in acute and chronic infections (3). Tissue catabolism during starvation can release Zn into circulation and increase PZC (3).

PZCs change after meals, during infections, inflammation, hemolysis, and under stress and trauma (22). Zn concentrations in plasma fluctuate during the menstrual cycle (23). Lower PZCs are measured in people with obesity, hyperinsulinemia, hypertension, hyperlipidemia, chronic inflammatory disease, and those undergoing surgery (24–26).

In summary, the latest consensus is that although not as consistent and reliable as biomarkers used for most medical conditions and some specific nutritional deficiencies, PZC is the best biomarker of population Zn status and predictor of functional responsiveness to Zn interventions in humans. It is a biomarker of exposure and of the risk of clinical Zn deficiency. PZC is a useful indicator of severe to moderate Zn deficiency and responds consistently to Zn supplementation. Nevertheless, PZC is less responsive when additional Zn is provided with food. Similarly, PZC predicts functional changes to Zn interventions only when initial PZC is very low. Furthermore, PZC does not necessarily reflect cellular Zn status due to very tight homeostatic control mechanisms that keep PZC within a narrow range. PZC on its own is not effective in assessing the impact of various dietary Zn interventions, particularly when the change in Zn intake is marginal, thus a more sensitive and specific indicator is needed to identify early-stage Zn deficiency states. The World Health Organization points out that the development of more suitable Zn biomarkers is still a high priority (26). Further research is required to identify and evaluate potentially more useful bioindicators of Zn status with increased sensitivity and specificity and responsive to modest changes produced by diet-based Zn interventions in humans.

## Evaluation of the accuracy and usefulness of available Zn status biomarkers

The latest publicly available review paper that evaluated the usefulness of existing biomarkers of Zn status in humans is the Biomarkers of Nutrition for Development (BOND) Zinc Review published in April 2016 (5). After reviewing Zn biomarkers, The BOND Zinc Expert Panel separated indicators into three classes: potentially useful (hair, urinary Zn, and neurobehavioral function) emerging (nail Zn, taste acuity, Zn kinetics, and Zn dependent proteins), and non-useful Zn biomarkers (erythrocyte and leukocyte Zn and Zn dependent enzymes). Shortly after, in September 2016, another review paper issued by Lowe (27) additionally assessed the potential and emerging biomarkers originally identified by the BOND.

Based on data from systematic reviews, the only other two recommended indicators, besides PZC, were dietary Zn intake and height-for-age of growing infants and children (5, 27).

Zn dependent enzymes and proteins, hair Zn concentrations, neurobehavioral function, markers of inflammation, taste acuity, DNA damage, and oxidative stress have been revised, and while some promising supporting evidence was shown, none of the biomarkers were endorsed, pointing out that further research is needed before any of these biomarkers can be used for evaluating Zn status of individuals or populations.

A systematic review is currently underway, updating the Biomarkers of Nutrition for Development (BOND)-Zinc Review, to determine which indicators accurately demonstrate changes in Zn status in response to Zn supplementation or depletion [Rasgado et al. (28) PROSPERO, CRD42020219843^1^ ] and once released, is expected to provide some new insights.

Back in 2016, the diagnostic performance of fatty acid desaturases 1 and 2 (FADS1/2) for predicting Zn deficiency/adequacy was not carefully assessed as there was a limited number of trials reporting on the proposed interactions, and none of the currently published human studies had been completed. Insufficient information on the activity of Zn-dependent desaturase enzymes was available at the time to draw robust evidence-based conclusions. Over time, an increasing body of evidence has accumulated to suggest that the linoleic acid: dihomo-γ-linolenic acid ratio (LA: DGLA) can be used as an additional, potentially more sensitive, biomarker of Zn status, both in animals and humans (29–35).

As shown by a recent systematic review FADS1 and FADS2 can be considered as candidate biomarkers for assessing Zn status and effectiveness of low dose Zn interventions, however additional research is needed to clarify the proposed associations and applicability of utilizing fatty acid desaturase activities as Zn status biomarkers while adjusting for all associated covariates and confounders (35).

This review summaries all potential currently identified confounders and covariates that should be taken into consideration when proposed relations are examined and presents a comprehensive list of recommendations for further research on this topic.

## The biochemical and dietary basis for the causal association between Zn and FADS1 and FADS2 activity

**FIGURE 1:**
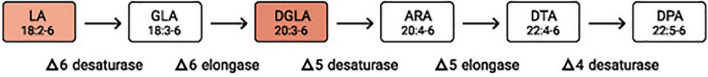
Metabolic pathway of n-6 fatty acids. Since the elongation steps are quick and the desaturation steps much slower, desaturases are regarded as the rate-limiting steps. LA, linoleic acid; GLA, γ-linoleic acid; DGLA, dihomo-γ-linolenic acid; ARA, arachidonic acid; DTA, docosatetraenoic acid; DPA, docosapentaenoic acid.

FADS1 (Δ5 Desaturase, D5D) and FADS2 (Δ6 desaturase, D6D) are membrane-bound desaturase enzymes involved in the synthesis of n-6 and n-3 sequence of polyunsaturated fatty acids from dietary linoleic acid and α-linolenic acid (36). Both enzymes are extensively expressed in the human tissues with the highest levels found in the liver (37). Delta 6 desaturase converts linoleic acid (18:2n6, LA) to γ-linolenic acid (18:3n6, GLA). GLA is then elongated to dihomo-γ-linolenic acid (DGLA, 20:3-6) by delta 6 elongase (Figure 1). Delta-5 (D5D) and delta-6 desaturases (D6D), coded respectively by FADS1 and FADS2 genes, are the rate-limiting enzymes for PUFA conversion. The Δ6-catalyzed step necessary for the conversion of LA to DGLA is the highest flux pathway, thus we propose that an increase in the LA/DGLA ratio could be a sensitive indicator of Zn deficiency (below presented findings).

**FIGURE 2:**
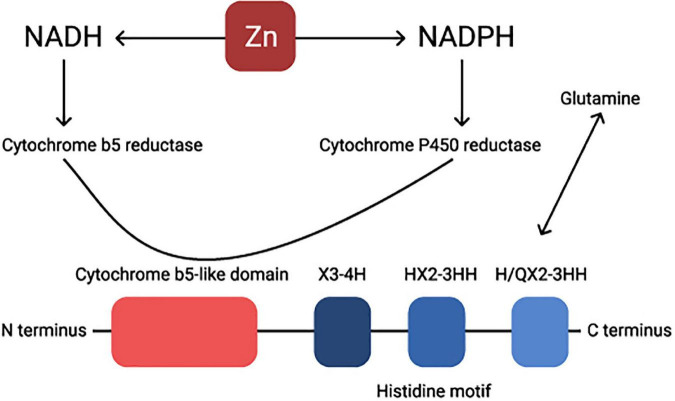
Visual presentation of the structure of a Δ6 desaturase enzyme. Zn regulates NADH-NADPH cycle. Cytochrome P-450 activity is considerably diminished under Zn deficiency. NADH, nicotinamide adenine dinucleotide hydride; NADPH, nicotinamide adenine dinucleotide phosphate-oxidase.

Structurally, these two enzymes are very similar, they both have an N-terminal cytochrome b5 domain-carrying heme-binding motifs, two membrane-spanning domains, and three histidine-rich motifs HX3-4H, HX2-3HH, and H/QX2-3HH (37, 38). The glutamine is essential for delta 5 and delta 6 activity and it usually replaces the first histidine-rich motif (Figure 2; 37). Human FADS1 and FADS2 genes are confined on chromosome 11, and both genes are composed of 12 exons and 11 introns. Considering the closeness of their promoters, the transcription of both genes in delta 5 and delta 6 desaturases are rate-limiting steps in the synthesis of PUFA as the enzyme catalyzes the addition of a double bond at the sixth carbon-carbon bond position from the carboxylic acid end in fatty acids (39).

Finally, NADPH reductase, an enzyme essential for FADS1/2 activity, is known to be Zn dependent (40, 41). Zn is an important, but not necessarily a unique and exclusive, cofactor in the metabolism of essential fatty acids (42) involved in at least two stages, conversion of LA to GLA and mobilization of DGLA to ARA (43, 44). Both delta 5 and delta 6 desaturases are represented as Zn-dependent enzymes (40, 44).

**FIGURE 3:**
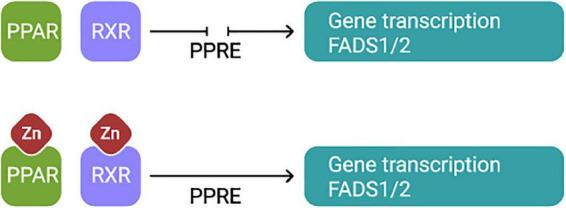
PPARs form a heterodimer with RXR and simultaneously they bind to the PPAR response element for transcriptional activation of FADS1 and FADS2 genes. Both, PPARα and RXR have in the DNA binding domain characteristics Zn fingers domains essential for appropriate function of the protein. Thus, Zn plays an essential role in the function of these transcription factors. PPARS, The peroxisome proliferator-activated receptors, RXR, Retinoid X receptor, PPRE, PPAR response element.

The factors affecting the expression of FADS1 and FADS2 are not entirely known. Some believe that the activity of desaturases may be affected by tissue-specific mechanisms that involve both pre- and post-translational actions (39). Others consider that the regulation is accomplished by the feedback control of the transcriptional regulation of fatty acid desaturase genes, facilitated *via* signaling pathways triggered by sensors inserted in cellular membranes, in response to environmental factors (45). FADS1 and FADS2 are target genes for proliferator-activated receptors for transcriptional regulation. Two Zn finger domains are crucial for the appropriate functioning of this protein (46; Figure 3). Zn deficiency causes modifications in proliferator-activated receptors signaling, and Zn treatments induce the expression of target genes (47).

Once it was noted that deficiencies of fatty acids and Zn presented clinically similar symptoms, a close association between the two was anticipated (43, 48). The potential link between impaired FADS activity and reduced metabolism of essential fatty acids caused by dietary Zn deficiency was initially shown in the early 1980s in animal models (44, 48–51). A low-Zn diet was associated with decreased activity of fatty acid desaturase enzyme 1 (FADS1) and lower integration of arachidonic acid into lipid subclasses signifying that FADS1 activity may among other factors, respond to slight alterations in dietary Zn intake (52). Over the years, this hypothesis was confirmed by others (30, 32–34, 53, 54).

In summary, findings from several studies imply that Zn deficiency or inadequate dietary Zn intake, among all other possible factors, could contribute to reduced activity of desaturase enzymes and that changes in FADS1 and FADS2 activity should be additionally examined as possible new markers for estimating Zn status.

## Potential covariates and confounders of Zn and FADS1/FADS2 interrelations

**FIGURE 4:**
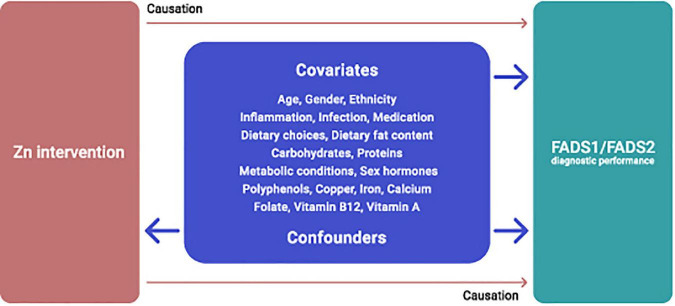
Potential covariates and confounders of dietary Zn intervention and FADS1/FADS2 interrelations.

The multifaceted roles of Zn and fatty acid enzymes imply that an interaction between Zn and FADS1 and FADS2 activity is almost certainly affected by various covariates and cofounders, most of which are yet to be determined and sufficiently and adequately examined. Currently available data indicate that dietary intake of macro and micronutrients, polyphenols, certain medical conditions, inflammatory conditions, provision of Zn in fasted or non-fasted states may affect the proposed Zn-FADS1 and FADS2 interrelations (Figure 4), suggesting that FADS activity may not be entirely specific for Zn status, an assumption that requires further research.

### Gender age and ethnicity

The role of gender, age, and ethnicity are additional factors that have not yet been studied in the context of Zn and essential fatty acid metabolic pathways. Lohner et al. (55) conducted a systematic review to explore gender difference in the long-chain polyunsaturated fatty acid status in human populations.

Analysis of data from 51 studies reporting fatty acid composition of plasma and erythrocyte membrane lipids and adipose tissue, revealed a higher contribution of the n-6 essential fatty acids, arachidonic acid, and docosahexaenoic acid to plasma total lipids and plasma phospholipids in women compared to men (55). There were no gender differences for linoleic (LA) and α-Linolenic acid (ALA). Higher activity of desaturases, mainly delta 6-desaturase contributed to the higher arachidonic acid (AA) and docosahexaenoic acid (DHA) values found in women (55).

Women have a higher capacity to synthesize DHA to LA than men, as shown by both animal and human stable isotope studies (56). Examination of desaturase activities in relation to gender in rats demonstrated that delta 5 and delta 6 desaturases activity was one to three times higher in females than in males. The delta 5-desaturase protein was higher and the mRNA expression of delta 5 and delta 6 desaturase genes was 3.8 and 2.5 times greater in females (56). Similar findings were provided by Guo et al. (57) lipid indicators differed between men and women in the Chinese adult population. However, contradictory findings were also reported, showing lower levels of delta 6 desaturase were measured in Swedish women (58). Burdge et al. (59) found no statistically significant differences in fatty acid composition among men and women.

The effects of gender and sex hormones on essential fatty acid metabolism in humans have been examined in depth by Childs et al. (60). Testosterone inhibits, while estrogen stimulates the transfer of essential fatty acids into their longer-chain metabolites (61). Loss of desaturase activity was reported due to reduced estrogen (62, 63). The use of hormonal contraceptives is also influencing fatty acid status (64), epigenetic modifications, i.e., DNA methylation, that affect desaturase activity differ among genders (65). While none of the presented findings directly confirm the influence of gender on fatty acid metabolism of people consuming Zn enriched diets, it clearly shows that gender-associated differences exist and should be taken into consideration. As stated by Lohner et al. (55) in studies reporting fatty acid composition in serum phospholipids, serum total lipids, or erythrocyte membrane lipids, gender distribution should be regarded as a significant potential confounding variable. Thus, gender differences in fatty acid status and hormonal regulation should be considered as covariates when examining the influence of Zn interventions on fatty acid metabolism.

The influence of aging on fatty acid composition has been investigated over the years. Delta 6 desaturase activity is shown to be reduced in the elderly compared to middle-aged healthy Tunisian subjects, by 10 and 24% in men and women (66). Decreased delta 6 activity due to aging has been described in animals (67) and humans (52, 68). In post-menopausal women, aged ≥51 years, the values of the n-6 polyunsaturated DGLA and AA were significantly higher in women compared to men of comparable age, while DHA values did not differ among the genders (55). In the age category, 13–50 years, out of all fatty acids assessed, DHA levels were considerably higher in women, while DPA values were significantly lower in women compared to men (55). Finally, as dietary habits are considerably affected by aging (69), an adequate assessment of dietary patterns is essential when interrelations among fatty acid levels, Zn status, and aging are examined.

Furthermore, significant ethnic differences in desaturase activities have been reported. A comparison of data from African Caribbeans, Asian Indians, and white Europeans revealed that delta 5 desaturase was highest in African Caribbeans, while delta 6 desaturase activity was lowest in Asian Indians (70).

Comparison of reported delta 6 desaturase levels among studies in White Caucasians (31) and Asians (71) demonstrate lower levels of delta 6 desaturases in Asians. There were statistically significant differences in FADS2 performance between Caucasian and non-Caucasian children, while no difference was reported for FADS1 activity (72).

There are several reports of differential effects of FADS polymorphisms on delta 5 and delta 6 desaturation indicators depending on ethnicity. Delta 5 activity was significantly lower in white Europeans relative to African Caribbean women.

Higher arachidonic acid levels have been described in people of Black African origin, showing ethnic differences in the FADS1 genotype (73). Ethnic-specific effects of FADS polymorphism on desaturase activity in Caucasians and East Asians were shown by (74). Overall, the lowest levels of delta 6 desaturase are reported in Asians and the highest in Africans. However, specific studies of ethnic differences in the FADS1 and FADS2 gene expression in respect to Zn status are yet to be conducted.

Estimated desaturase activities differ among metabolically healthy and unhealthy individuals, lower delta 6 desaturase, and to some extent lower delta 5 activity, were reported in people with an elevated risk of cardiovascular diseases, diabetes, obesity (75). There was a direct positive association between the markers of obesity and FADS2 activity and an inverse association with FADS1 in a Swedish study, with relationships being independent of body mass index and physical activity (58). Delta 5 desaturase activities were also shown to be inversely related to metabolic dysregulation (76, 77). FADS1 activity was significantly associated with an increased risk of type 2 diabetes in Japanese population (78).

Additionally, a significant number of studies to date have confirmed the link between dietary Zn intake/Zn status with obesity, hyperinsulinemia, hypertension, hyperlipidemia, chronic inflammatory disease, cardiovascular diseases (24–26). It is important to note that even in these instances when disturbed metabolic activity is present, Zn status was directly inversely associated with delta 6 desaturase activity (33, 34, 79). However, what remains unclear, and certainly requires further investigation, is whether dysregulation of desaturases, usually coupled with reduced Zn levels, is a reason for or a result of metabolic disturbances. As these factors are closely interrelated, the extent to which desaturase activity acts as specific biomarkers of Zn status and metabolic health requires further investigation.

### Dietary components

Dietary habits have a major influence on the fatty acid composition in tissues (80). All classes of macronutrients (fats, proteins, and carbohydrates) as well as some minerals and other dietary components including polyphenols, have been shown to influence mRNA expression and activity of desaturase and elongase enzymes (81) which suggests that FADS1 and FADS2 are not exclusively responsive to modifications in dietary Zn intake and that FADS activity is not specific to Zn status.

The fat content of diets has been shown to be an important element in fatty acid metabolism, both in terms of quantity and composition, as demonstrated by both animal and human model studies (82, 83). Repressed activities of delta 5 and delta 6 desaturase and elongases were seen in animals on high fat diets (84, 85) a tissue-specific regulation of desaturase activities in response to high-fat diets has been reported (86).

Likewise, a suppressed delta 6 desaturase activity was shown when the effect of provision of high-fat diet with 40% of fat was compared to a low-fat diet, 20% fat, in a crossover human trial in post-menopausal women (83). Furthermore, inconsistent findings are found on the effect of diets deficient in essential fatty acids; both increased (87–90) and decreased (91, 92) delta 5 and delta 6 desaturase and elongase activities were reported. Similarly, diets enriched in polyunsaturated fatty acids are shown to reduce the activity of delta 5 and delta 6 desaturases (93). Diets rich is saturated fats and cholesterol tend to suppress activities of both desaturases (85, 94–97).

The available evidence demonstrates that both the quantity of fat as well as the fatty acid composition influence the activity of desaturases. Appropriate quantitative assessment of dietary fat intake during Zn intervention trials is necessary to ensure that any observed changes in the activity of desaturases are not confounded by differences in dietary fat intake. Each of the individual factors, i.e., the amount of fat, as well as the type of fat, represent important confounding factors between Zn-fatty acid relations and should be carefully examined. Tissue-specific regulation of desaturases is another important aspect to be considered.

Some authors propose that measurement of fatty acid composition of blood lipids and adipose tissues can augment dietary assessment methodologies (98). In addition, it was shown that even though red blood cells lack desaturases and elongases, the fatty acid composition of their membranes reflects liver synthesis, and as it is less affected by variations in fatty acid intakes it should be used in preference to plasma/serum fatty acid ratios (99, 100). The rate of changes in the fatty acid composition of fatty acid in red blood cell membranes is slower than that seen in plasma lipids (101).

The relation between fatty acid metabolism and dietary carbohydrates has been examined over the years, providing consistent evidence of increased FADS2 expression in response to high carbohydrate diets in male rats (102) and in mice (103). The quantity of carbohydrates matter, for example, provision of sucrose, 30%, lead to reduced delta 5 desaturase activity (104, 105) with no changes in delta 6 enzyme activity (105). In contrast, 62.5% sucrose rich diet increased both FADS1 and FADS2 hepatic gene expression (106).

In addition, high protein diets/extra protein intake stimulate desaturase activity. Increased delta 6 desaturase action was measured in rats fed an excess protein diet, 35% of protein, vs. rats on high protein diets with 25% of protein (87). The type of protein consumed is also important, casein fed rats reported to have improved microsomal delta 6 activity (107). Supplementation with L-cysteine, L-glycine, and L-methionine increased delta 6 activity even further, with the highest effect being observed with the addition of L-methionine (108). In conclusion, it seems that FADS1 and FADS2 expression is influenced by dietary protein, and additionally by specific amino acids, but this requires further examination.

Polyphenols can affect fatty acid synthesis. Increased FADS1 and FADS2 mRNA expression was measured in HEpG2 cells supplemented with resveratrol (109). Liver estimates for delta 5 and delta 6 desaturase activities were increased in anthocyanin supplemented group of rats (110). FADS2 gene expression was higher in chickens supplemented with isoflavone compared with controls (111).

In addition to Zn, desaturases are shown to be affected by other minerals. For example, a calcium-deprived diet provided for 60 days to rats produced a 45–55% decrease in delta 6, and a 30% inhibition in delta 5 desaturase activity, correspondingly (112), impaired delta 6 and delta 5 desaturase activities were seen in the liver of rats supplied with suboptimal iron levels (113, 114). Data from iron deficient individuals support the findings, iron deficient diets impair activity of both desaturases (115, 116).

Given the widespread occurrence of low calcium intake and iron in low- and middle-income countries, the potential implications of this finding should not be overlooked and deserves further research. Copper has also been shown to affect fatty acid metabolism (117). Modified Cu/Zn ratios in the plasma were associated with an altered fatty acid profile in subjects with dyslipidemia, the Cu/Zn ratio was directly linked to alpha-linolenic acid (33). Likewise, Cu/Zn ratio was directly correlated with elongase activities in hemodialysis patients (79). Increased delta 6 desaturase activity was observed with the addition of Cu (33, 118, 119).

The plasma Cu/Zn balance is altered in many disease states; a disturbed Cu/Zn ratio may be an active modifier of the LA/DGLA ratio and desaturase activities, so an examination of Zn-fatty acid relationships in unhealthy cohorts should encompass the analysis of both nutrients.

A dose dependent increase in FADS2 expression was seen in rats supplemented with retinoic acid (120). Zolfaghari et al. (121) reported decreased FADS1 expression in rats on diets with 4 mg of retinol compared with rats consuming vitamin A deficient diets. Vitamin A increased hepatic phospholipid activity of both desaturase enzymes (122). A few studies that examined the effect of folate/vitamin B-12 on desaturases provided ambiguous results, yet they indicate that these vitamins may have a role to play in the process and they should be taken into consideration in further research (123, 124).

## FADS1, FADS2, Zn intake and Zn status interrelations–animal experiments

The effect of Zn deficiency on FADS1/2 performance was initially examined in the early 80s by Cunnane and Wahle (125), Clejan et al. (49), and Ayala and Brenner (44). Consistent findings were reported, Zn deficiency contributed to reduced activity of both enzymes, delta 5 and delta 6 desaturase.

Cunnane and Wahle (125) were the first to demonstrate that Zn modulates linoleic acid metabolism in rat mammary glands, by modifying the desaturase activity of microsomes. In their experiment, 38 Sprague-Dawley rats were provided either a purified Zn-supplemented or a Zn-deficient diet for 6 weeks, and the effect of Zn deficiency on the fatty acid composition of plasma lipids and microsomes of the liver, intestine, and testes were explored. Among the polyunsaturated fatty acids, DGLA was significantly reduced in the rats consuming the Zn-deficient diet. The Zn-depleted rats also had a 25% reduction in delta 6 desaturase activity in liver microsomes, while delta 5 desaturation was decreased by 53%. Furthermore, Zn-deficient rats had hypertriglyceridemia, and Zn supplementation restored serum triglycerides levels to normal which demonstrates a strong physiological link between Zn and essential fatty acids and demonstrates that Zn deficiency might be accountable for attenuated desaturase activities (125).

Similar findings were provided by Ayala and Brenner (44) who assessed the influence of Zn on desaturating enzymes of liver and testes microsomes and their effect on fatty acid and lipid modifications of tissues using male weaning Wistar rats. Zn-adequate (55 ppm of Zn) or Zn-deficient diets (1.2 ppm of Zn) were given to rats for 60 days.

A decrease of essential fatty acids of the linoleic family in plasma was apparent after only 18 days, which implies that Zn deficiency could produce a rapid change in desaturase activity (44).

Zn deficiency contributed to modifications in both FADS1 and FADS2 activities but to a different extent. The identical level of Zn deficiency produced a 45% decrease in FADS2, while FADS1 action was entirely diminished (44). However, when the interaction between Zn deficiency and desaturase activity was examined in the presence of different types of dietary fats (coconut, linseed, and sunflower oils), Zn deficiency had no negative effect on desaturases action (48, 126, 127). It seems that the type of dietary fats affects the desaturase actions. Fat-free diets improve the activities of desaturases considerably, while diets with high levels of polyunsaturated fatty acids suppress them.

The idea that the impact of Zn deficiency on lipid metabolism may be affected by the type of dietary fat consumed was also demonstrated by Waldhauser and colleagues in 1999 (128). Four groups of rats were fed Zn-adequate (45 mg Zn kg^–1^) or Zn-deficient (0.5 mg Zn kg^–1^) diets with olive oil or linseed oil as the source of fat. The rats were force-fed by gastric tube for 13 days to ensure comparable food intake. The results confirmed that Zn deficiency impacts the metabolic balance of n-3 and n-6 polyunsaturated fatty acids. In the rats fed linseed oil, Zn deficiency produced a marked increase in the ratio between n-3 and n-6 polyunsaturated fatty acids in liver phospholipids, while in the rats fed olive oil, Zn deficiency had only slight effects on the fatty acid composition of the liver phospholipids. Similarly, the composition of dietary fat affected only hepatic delta 6 desaturase enzymatic activity (128).

In addition, the consumption of an essential fatty acid-deficient diet is shown to be paralleled by a similar rise in the hepatic abundance of FADS2 and the increase in hepatic delta 6 desaturase activity (52) whereas delta 6 desaturase action was very low in non-hepatic tissues (129). However, when essential fatty acid deficiency is of metabolic origin, i.e., caused by Zn deficiency, desaturases activity is reduced (50). Finally, the inhibition of the desaturases by Zn deficiency is intense and produces a more rapid decline in tissue arachidonic acid and docosahexaenoic acid than does the immediate dietary deficiency of all the omega 6 or omega 3 polyunsaturated fatty acids (130).

To clarify inconsistencies and confirm that the effect of Zn deficiency was not mistakenly misconceived by low food intake, Eder et al. in 1995 examined the role of Zn in desaturase activity by a series of experiments with Zn-deficient rats using a force-feeding technique that ensures equal food intake between the intervention groups.

In Zn-deficient rats fed a diet comprising of 5% safflower oil lower levels of total polyunsaturated fatty acids were measured than in rats fed a Zn-adequate diet. The findings were clear, Zn status had an evident role in delta 5 and delta 6 desaturation in subjects with appropriate food and energy intake. Furthermore, in animals fed fat-free diets, the effect of Zn deficiency on delta 6 desaturation activity was even more noticeable (48).

When the role of Zn on delta 6 desaturase activity was evaluated using a chicken (Gallus gallus) model comparable findings were provided, the concentration of DGLA decreased and the LA/DGLA ratio increased in animals with lower dietary Zn intakes (30, 31, 33). Once Zn-adequate control (42.3 μg Zn g^–1^) or Zn-deficient diets (2.5 μg Zn g^–1^) with identical fatty acid content were provided to birds for 4 weeks the expression of hepatic delta 6 desaturase was notably higher in the control group, and the LA/DGLA ratio was elevated in the low Zn compared to the control Zn group, 22.6 ± 0.5% and 18.5 ± 0.5% w/w, correspondingly.

The erythrocyte LA/DGLA ratio differentiated Zn status among Zn-adequate and Zn-deficient subjects. Furthermore, differences in the LA/DGLA ratio were evident within 7 days, signifying that the ratio can show changes in the dietary Zn status quickly and can detect early stages of Zn deficiency/inadequacy that generally, due to the lack of apparent symptoms stay unrecognized.

Similar findings were reported when the efficacy of the LA/DGLA ratio to predict the Zn status of animals consuming Zn biofortified wheat-based diets was examined (33). Two groups of birds (*n* = 15) were fed two different diets, a “high-Zn” diet (46.5 ppm Zn) and a “low-Zn” diet (32.8 ppm Zn), for 6 weeks. The expression of hepatic delta 6 desaturase had lower mean values and consequently the erythrocyte LA/DGLA ratio was higher in birds fed low-Zn diets.

A 14-ppm differential in dietary Zn content was sufficient to detect differences in the LA/DGLA ratio among the groups, which additionally confirms the sensitivity of the marker to respond to changes in dietary Zn intake (33). Serum Zn concentrations of the birds were also measured, in both studies, higher values were reported in the Zn control versus the Zn-deficient diet group of birds (30, 32). There was a relative increase in gene expression of the cytokines: interleukin 1 beta (IL-1β), interleukin-6 (IL-6), and tumor necrosis factor-alpha (TNF-α) in the control group. Other measured parameters, i.e., Zn transporters (i.e., Zip6, Zip9, ZnT1, ZnT5, ZnT7); transcription factor: nuclear factor kappa B (NF-κB); brush border enzymes: Na + K + ATPase, sodium-glucose transport protein 1 (SGLT-1), aminopeptidase, sucrose-isomaltase, and binding metallothionein-4 protein (MT4) were not noticeably different between the groups in the first study, while a higher mean value in the tissues collected from the birds fed a low-Zn diet was observed in the second study. Longer study duration may be an explanation, as longer period is needed for the detection of changes in gene expression of various Zn transporters. The expression of the hepatic FADS2 gene was investigated in both studies and demonstrated a significant alteration in delta 6-desaturase gene expression in the experimental group with higher dietary Zn intakes (30, 33). Comparable data were provided by Beasley et al. (131), a reduced LA/DGLA ratio was measured in birds after 2 weeks of consumption of nicotianamine enriched Zn and Fe biofortified wheat-based diets.

Finally, consistent findings were reported in humans, the concentration of plasma DGLA was decreased and the LA/DGLA ratio was increased in apparently healthy people with lower dietary Zn intakes (32). However, while there was a significant difference in DGLA production and the LA/DGLA ratio between the groups, the PZC stayed unchanged, probably due to the effective homeostatic regulation of Zn absorption. Additionally, plasma/serum Zn may not be sufficiently sensitive to detect relatively small differences in dietary Zn intakes, compared to the LA/DGLA ratio.

## Human studies–uncontrolled nutrient (dietary) intervention and human randomized controlled trials

The NHANES study involving more than 1,500 participants demonstrated a negative correlation between serum LA/DGLA ratio and serum Zn status, with statistical significance seen in men only (132). However, while correlations of the LA/DGLA ratio with Zn intake adjusted for energy intake were not statistically significant, they showed negative associations between the assessed parameters in women, while a positive interaction was found in men, which points out that gender differences may have a role to play. Finally, the provision of foods rich in Zn and poor in polyunsaturated fatty acids was adversely associated with serum LA/DGLA ratio (132).

In a 10 weeks Zn controlled feeding trial conducted on thirty-six healthy adult men, 18–51 years of age, in participants who consumed Zn biofortified wheat bread (1.6 mg/day of additional Zn) for 6 weeks increased FADS2 and decreased FADS1 activities were reported, with no changes in plasma Zn concentrations, DNA strand breaks or blood glutathione concentrations (35). This clearly shows that the activity of desaturase enzymes is much more sensitive to subtle changes in dietary Zn intakes than all other measured parameters.

Comparable findings were reported by Suh et al. (133), dietary Zn modulates the metabolic pathway of lipids. No changes in PZC but alterations in lipid metabolism were seen in eighteen 19–50 years old men exposed to Zn biofortified rice. FADS2 activity was increased, by 56%, during the provision of diets containing 6 mg of Zn/day and 1.5 g of phytate for 2 weeks. A considerable, 126%, increase in FADS2 activity was reported when a diet containing 10 mg Zn/day with no phytate was provided for 4 weeks to this group of men (133). On the other hand, FADS1 activity was decreased by 29 and 45.6% during the provision of two diets, correspondingly. The activity levels of both desaturases returned to baseline following the provision of 25 mg Zn/day for 3 weeks.

Similar negative correlations between the LA/DGLA ratio and dietary Zn intake and Zn status were also seen in people with underlying chronic conditions. Takic et al. (79), reported a negative correlation of the ratio with both dietary Zn intake and serum Zn status in 40 Serbian adult patients undergoing hemodialysis. A cross-sectional study conducted in China on 232 community-dwelling subjects, 35–60 years of age, with hypertension described an indirect association between delta 6 desaturase activity and serum Zn levels (71). Yari et al. (134) described lower delta 6 and higher delta 5 desaturase activity in type 2 diabetes middle age and older men patients with lower serum Zn concentrations, pointing out that higher serum Zn concentrations were associated with a lower risk of developing type 2 diabetes. Finally, a prospective study performed on 661 men, 42–60 years of age, demonstrated that higher delta 5 and lower delta 6 desaturase activity was associated with higher serum Zn concentrations, and a lower risk of developing metabolic syndrome (135).

Besides observational studies, the effect of Zn interventions/Zn deficiency on fatty acid enzyme activity was additionally examined in randomized controlled trials (17, 34, 100). A 20-week, double-blind randomized controlled trial conducted in 186 school-age Beninese children, provided with water filtered with Zn fortification or a placebo chamber, demonstrated that Zn status plays an important role in fatty acid desaturation. At the baseline, plasma Zn concentration was directly correlated with DGLA and LA/DGLA ratios, and Zn deficiency lead to an interruption of delta 6 desaturase activity, hindering the conversion of LA into DGLA (34).

On the other hand, no similar effect was observed for delta 5 desaturase activity which indicates that FADS1 expression may not be affected by dietary Zn intake and Zn status, or at least, not under certain conditions.

Contrary to previous findings, no statistically significant differences in the LA/DGLA ratios were reported in groups of children receiving either a daily portion of Zn-fortified, filtered water with an average 2⋅8 mg Zn/d, or non-fortified filtered water, mean dietary Zn intake was 8.1 mg/day (34).

However, increased Zn intake for 20 weeks significantly decreased ALA and prevented the reduction of nervonic acid, a longer chain n-9 monounsaturated fatty acid, in plasma total phospholipids.

This means that Zn, besides affecting desaturase activity and n-6 fatty acid composition, may also influence the activity of the fatty acid elongases in the n-9 synthesis pathway which requires further investigation.

A recent double-blind randomized controlled trial found no changes in FADS1 nor FADS2 activities in Bangladeshi preschool children, fed Zn biofortified rice diets (providing 1 mg of Zn/day) for 9 months (18). Several reasons might explain the lack of interaction between desaturases activity and dietary Zn intake. Children were severely Zn deficient and stunted, and as in these situations, infections and inflammation are frequently found, thus these factors may have confounded the interpretation of the outcome measures. Besides, the additional dose of 1 mg of Zn/day might not have been sufficient to result in changes in desaturase performance even in presumably zinc deficient children.

Analogous findings were provided when the efficacy of Zn biofortified wheat in improving Zn status of consumers was assessed by an individually randomized, double-blind, placebo-controlled cross over study conducted in fifty Pakistani households (136). Although a significant increase in PZC levels were seen after 4 weeks, no changes in PZC and desaturase activity levels were present after 8 weeks of dietary intervention. Low dietary Zn intake contributes to reduced FADS activity, but no statistically significant associations were achieved (136).

Several facts could explain this lack of significant association; the short duration of the intervention, inability to measure inflammatory markers and adjust PCZs accordingly, the likely presence of inflammation (as assessed by Cu to Zn ratio), and the fact that the study had a relatively small sample size and was not powered to detect variations in FADS activities.

The effect of a 24-month Zn supplementation (30 mg elemental Zn) on membrane fatty acid composition was investigated in patients with type 2 diabetes. The study revealed an increase in the abundance of polyunsaturated fatty acids and improved flexibility of red blood cell membranes (48). The enzymatic activity of the delta 5 and delta 6 desaturase was unchanged by Zn supplementation. However, the arachidonic acid abundance was greater in the Zn supplemented group, which shows an increased enzyme activity, which most likely was not captured by an indirect method employed for evaluation of desaturase activity. Gene expression of FADS1 and FADS2 were also assessed, showing an increased expression of FADS1 gene in months 12 and 24 with respect to baseline in the Zn supplemented group, while FADS2 gene expression was similar in Zn and the placebo groups (100). The study findings suggest that Zn affects fatty acid composition by modulating delta 5, instead of delta 6 desaturase activity.

Finally, experimental data imply that Zn metabolism in the human body is influenced by food consumption, as Zn absorbed with food ends up in the liver *via* the portal circulation, while Zn taken without the food is delivered to peripheral blood plasma (137). As the metabolism of essential fatty acids occurs hepatically, the direction of Zn to the liver may additionally stimulate Zn contribution to fatty acid metabolism. Differences in the effect of Zn supplementation on FADS1 and FADS2 activities and plasma Zn concentrations were thus examined for the same chemical form and amount of Zn supplements provided in the fasted and non-fasted states for 2 weeks in apparently healthy men, 15–50 years old, living in California (17).

As anticipated, increases in plasma Zn concentrations were seen only when Zn supplements are taken without a meal, and Zn supplementation affected fatty acid desaturation only when supplemental Zn was provided with food (17).

There was a statistically significant difference in FADS1 activity among participants consuming Zn with breakfast compared with those taking Zn in the fasted state, while FADS2 activity did not differ between the examined groups. Consumption of Zn with a meal or without it seems to affect changes in FADS1/FADS2 activities and should be examined further.

## Additional potential and emerging Zn biomarkers

Besides LA/DGLA ratio, some other biomarkers have emerged as potentially useful and have been tested to a degree over the recent years, i.e., Zn dependent proteins, nail Zn concentrations, oxidative stress, DNA integrity, erythrocyte and leukocyte Zn concentrations, glutathione concentrations, Zn kinetics, and taste acuity; however, there is still unconvincing evidence for their use as Zn status indicators (21, 29).

Zn levels of platelets, granulocytes and lymphocytes were more efficient in revealing Zn depletion conditions than plasma Zn levels, pointing out that cellular Zn levels may be more useful Zn status indicators (138, 139). Thymulin and gut microbiota composition are additional indicators proposed as possibly suitable to be employed as accompanying biomarkers of Zn status. Recently, Cheng et al. (140) presented Zn status index (ZSI) model, which is based on three different indicators: LA/DGLA ratio, mRNA expression of Zn associated proteins and gut microbiome composition.

### Response of thymulin to changes in Zn intake

Thymulin, also known as a thymic hormone, is a hormone required for the differentiation and development of T helper cells. It is engaged in T-cell differentiation and improvement of T and NK cell actions (141). The amino acid structure of this non-apeptide is the following: Pyr-Ala-Lys-Ser-Gln-Gly-Gly-Ser-Asn. Thymulin requires Zn for its biological activity, and as such, it has been suggested as a biomarker of Zn status (139).

The binding of Zn is linked to the biological activity of thymulin. Two forms of thymulin exist, the first one, with Zn, is biologically active, and the second one, deprived of Zn, is biologically inactive (139, 142). The thymulin-Zn connection was examined *in vitro* and *in vivo*, using different models of mild Zn deficiency, both in animals and in humans. Insufficiency of Zn reduced serum thymulin activity, which was corrected with a provision of Zn supplementation both in animals and humans (139, 142–144). Serum thymulin activity declined after 8–12 weeks following the introduction of a Zn restricted diet (139). Zn stimulated thymulin secretion from human thymic epithelial cells *in vitro* (145). Findings were supported by others, reduced serum thymulin levels were measured in Zn deficient mice (142, 146) and man (139, 147). Comparable data were shown in patients with underlying chronic health conditions, sickle cell anemia, Chron’s disease, in patients with chronic renal failure and young cancer patients (139, 148, 149). same findings, of diminished thymulin activity, were reported for three different Zn deficiency model studies in humans: in subjects with dietary Zn deficiency, Zn deficient adult sickle cell disease patients, and mildly Zn deficient medical students. In all these subjects, Zn supplementation is shown as beneficial in restoring thymulin action (139). Thymulin from Zn deficient subjects included less Zn on a molar basis than thymulin from Zn sufficient subject, demonstrating that an inactive form of the peptide is not a cause for thymulin inactivity *per se* (139).

In addition, thymulin inactivity was also linked to reduced IL2 activity and changes in lymphocytes subpopulation (decrease in T101- and increase in T4 + and T4 + /T8 + cells).

Recently, DiSilvestro et al. (150) showed that in rats fed low Zn diets serum thymulin activity was reduced by 61%, while serum Zn levels were decreased by 31%; thymulin is shown as more sensitive to inadequate Zn intake than serum Zn. Further research is certainly needed to elucidate the entire role and potential limitations of this indicator. It would be beneficial to compare the sensitivity of FADS1/FADS2 gene expression with thymulin activity to determine which of the biomarkers is more receptive to changes in dietary Zn intakes.

### Response of the gut microbiome and Zn transporters to changes in Zn intake

Zn is essential for the proper activity of certain gut bacterial strains, and consequently Zn deficiency alters the composition of Zn-dependent microorganisms (151, 152). Low dietary Zn intake affects gut microbiota composition, decreases the production of short-chain fatty acids, and alters the metagenomic potential of the microbiota (152).

Increased abundance of Firmicutes and Proteobacteria was reported by Beasley et al. (131), while fluctuations in bacterial abundance of Clostridiales and Dorea genera (152) were shown in the group of subjects on high Zn diets. Even though different strains dominated, lower dietary Zn intakes were always coupled with alterations in the gut bacterial composition and reduction in the formation of short-chain fatty acids. Zn biofortified foods have a considerable beneficial effect on gut bacterial composition, their consumption lowers the abundance of potentially pathogenic bacteria, reduces inflammation, and stimulates the formation of short-chain fatty acids producing bacteria (153).

Gene expression of Zn transporters is influenced by dietary Zn deficiency, both decreased and increased expression has been demonstrated (33, 152, 154, 155). Zn transporters were significantly down-regulated in response to low Zn diet, but only in studies run for longer periods, i.e., 6 weeks (33). Likewise, delta 6 desaturase gene expression was down-regulated in experimental groups with lower dietary Zn intakes (30, 33).

Recently a Zn status index was created as a potential indicator of Zn physiological status. The model is based on three predictive elements of Zn status: LA/DGLA ratio, microbiome analysis and Zn-related gene expression (140). Although it looks promising, as it reveals different levels of Zn inadequacy or adequacy, it requires further testing, in larger, more distinct population groups, both in healthy and unhealthy cohorts. Finally, the validity of the model to predict Zn status/efficacy of dietary Zn interventions in humans is needed.

## Conclusion and recommendations for further research

An indicator that uniquely defines Zn status in different populations under various physiological conditions is still missing. The multifaceted role of Zn in numerous processes and pathways in the human body implies that one single biomarker may not be sufficient and that a set of Zn indicators may be necessary to recognize different Zn deficiency states among various populational groups.

FADS1/FADS2 gene expression could be used as an additional physiological indicator of Zn status in humans. New findings are very encouraging as they demonstrate that activity of desaturases, specifically measured by the LA/DGLA ratio, responds rapidly to changes in dietary Zn intake, both in animals and humans.

The erythrocyte LA/DGLA ratio responds to dietary Zn manipulations, within one to two weeks. In addition, the biomarker can detect early stages of Zn deficiency that usually, due to the lack of evident signs and symptoms, pass unrecognized.

Indeed, further studies and dietary intervention trials are needed to fully describe the effectiveness of this indicator and determine its overall specificity to act as a biomarker of Zn status. The activity of desaturases should be further examined in different settings, controlling for potential confounders in Zn deficient populations and population groups with a variety of Zn deficiency levels/stages. Further assessment of the relationship between usual dietary Zn intake and FADS1/FADS2 diagnostic performance is needed. It would be useful to measure the FADS1/FADS2 response after controlled manipulations of dietary Zn intake, conducting Zn depletion/repletion studies.

In addition, the initial activity of desaturases between individuals who do or do not appear to have a functional response to modification in their Zn intakes should be examined. It would also be valuable to compare FADS1 and FADS2 gene expression between individuals with clinical signs generally recognized as functional outcomes of severe Zn deficiency and contrast the findings with the results obtained in those with marginal Zn deficiency or Zn sufficiency (at least no deficiency *per se*). Furthermore, identification of factors that distinctively impact desaturases activity will expand our understanding of the age, sex, and ethnic-specific characteristics of fatty acid intake and metabolism and its relationship with Zn intake/status.

Additional randomized controlled human dietary Zn intervention trials are required to clarify alterations in desaturase activities in the presence of infectious and inflammatory conditions. Further research is needed to explain if dysregulation of desaturases activities is a reason or a result of metabolic disturbances in unhealthy population cohorts, and to what extent, commonly seen reduced Zn levels contribute to it. Further primary or secondary data analysis from controlled feeding studies are needed to clarify the impact of other minerals on desaturases, specifically the effect of calcium, iron, and copper deficiency and supplementation.

Likewise, the enzymatic activity of hepatic desaturases should be distinguished from the expression and activity of desaturases in non-hepatic tissues to define the exact role of dietary fats in desaturases activity and FADS1 and FADS2 gene expression. Inconsistent responses of delta 5 and delta 6 desaturases to Zn intake should be clarified, particularly the identification of other factors that may also change the kinetics of desaturase enzymes. Finally, FADS1/FADS2 gene expression could be matched and examined in relation to modifications of Zn-dependent proteins and genes at the main sites of Zn absorption.

Further studies are needed to determine the threshold dose of Zn (if such exists) that is needed to be taken with or without the food to influence the activity of desaturases and FADS1/FADS2 gene expression. The amount and form of Zn and the way it is given, in a fasted state or with food, should be clearly defined in Zn intervention trials before desaturase activity is to be determined.

Red blood cells membrane lipids seem to be the best choice for measuring conversion pathways as red blood cell fatty acid structure is unaffected by fasting status and is more stable over time, but simultaneous comparison of methods in an experimental design is needed to confirm this.

More research is needed to better describe the effectiveness of desaturases as accurate and precise indicators of variations in bioavailable Zn intake over time, i.e., high vs. low Zn intake and over short vs. long provision times. Dietary fat content, particularly intake of polyunsaturated fatty acids, should be assessed during Zn intervention trials to exclude potential confounding effects intake of fat may have on Zn-fatty acid relationships. Well-controlled timing of food in relation to Zn intake may help clarify metabolic effects/fatty acid alterations of dietary Zn intake and eliminate the potentially confounding effect of dietary patterns on assessed outcomes. Numerous dietary components can influence desaturase and elongase activities and thus, careful consideration of all dietary components is needed. Appropriate dietary data collection is of crucial importance in this instance. Further investigation should be pursued to elucidate current inconsistencies in terms of both, the quantity of certain macronutrients and their composition. The influence of other dietary components, i.e., polyphenols on desaturases activity should be additionally explored.

The role of aging, sexual development stage in women, gender in nutritionally at-risk life cycle stages, and ethnicity should be, whenever possible, taken into consideration and additionally examined with respect to relationships between desaturase activities and Zn interventions. An appropriate randomization process and strictly defined study entry criteria are necessary to eliminate both recognized and unrecognized covariates and confounders. Additional interventional and controlled feeding studies should investigate other possible interindividual factors that might affect the FADS1 and FADS2 performance, and the replicability, validity, sensitivity, specificity, ability to identify changes and suitability of a Zn biomarker for a population under study should be carefully assessed.

Finally, as a panel of biochemical markers of Zn is most likely needed to accurately determine Zn status, further work should aim to identify the most cost-effective combination of prospective indicators that could be used in low to middle income countries for predicting the Zn status of individuals and diverse populational groups, both healthy and unhealthy cohorts.

## Author contributions

MK performed the literature search, collected and analyzed the data, and wrote the manuscript. EB provided constructive feedback. Both authors approved the final version of the manuscript.

## Footnotes

1
https://www.crd.york.ac.uk/prospero/display_record.php?ID=CRD42020219843
